# Supplementary material for: The Impact of Implementation of Palliative, Non-Operative Management on Mortality of Operatively Treated Geriatric Hip Fracture Patients: A Retrospective Cohort Study
Source: J Clin Med. 2024 Mar 29;13(7):2012. doi: 10.3390/jcm13072012 (PMC11012274; doi:10.3390/jcm13072012)
Supplement: Supplementary file 1 [file jcm-13-02012-s001.zip › jcm-2918583-supplementary.pdf]

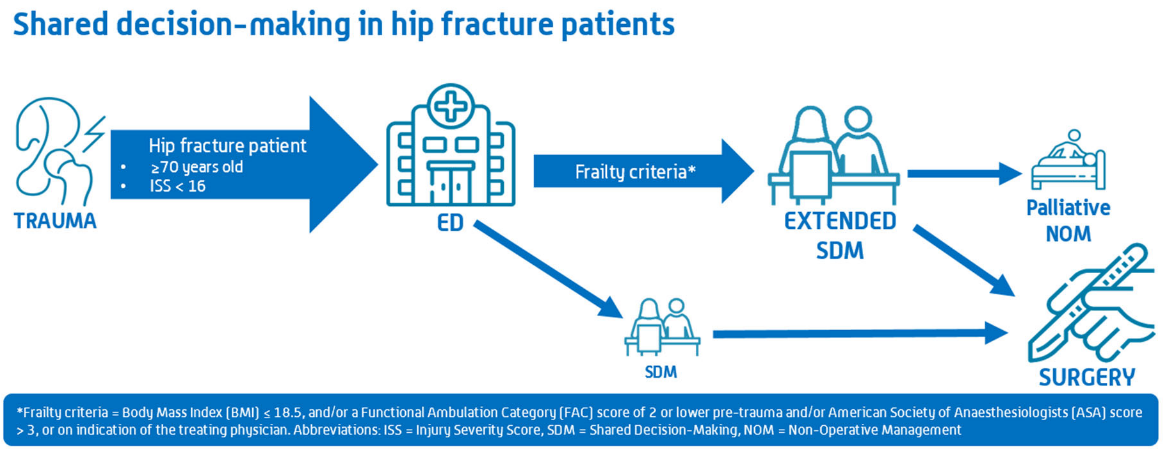

Figure S1. Renewed hip fracture pathway for geriatric patients.
